# Two different cases of SLE with neuropsychiatric manifestation

**DOI:** 10.1186/1546-0096-9-S1-P250

**Published:** 2011-09-14

**Authors:** V Panaviene, S Rusoniene, M Jakutovic

**Affiliations:** 1 Clinic of children’s diseases, Vilnius University, Lithuania; 2 Vilnius University children hospital, Lithuania

## Background

Juvenile Systemic Lupus Erythematosus (SLE) is an autoimmune disease with variable clinical features. Neurologic and psychiatric symptoms are reported to occur in 10 to 80 percent of patients either prior to the diagnosis of systemic lupus erythematosus, or during the course of their illness. The neuropsychiatric manifestations of SLE are varied, and may be classified as primary neurologic and psyciatric disease (eg, related to direct involvement of the neuropsychiatric system), and secondary disease (eg, related to complications of the disease and its treatment).

## Objective

Describe and compare clinical and laboratory features of two girls with SLE and neurologic manifestation.

## Methods

We report 15 and 10 years girls cases with SLE and similar neurological symptoms, MRI changes, but differrent origin of encephalitis.

## Results

First girls neurological symptoms (headache, depresssion, irritability) dated at the first onset of lupus. Neuropsychiatric symptoms manifestated, partial seizures occured. Immunological tests showed a very high titres of ANA, antidsDNA. The presence of a combination of anti-cardiolipin/beta2-glycoprotein I antibodies strongly associated with CNS vasculitis, abnormal MRI. Treatment with antiepileptic drugs, of steroids puls-therapy with further oral prednisone and intravenous cyclophosphamide were efective in reducing symptoms. Second girl’s analogous neuropsychiatric manifestaion occured after one year of lupus treatment with oral prednisone and after second cyclophosphamide puls, due to secondary factor – viral infection (EBV, HSV) with dominated organic dysfunction of the central nervous system (seizures, tetraplegy, pseudobulbar paralysis). Her antiphospholipid antibodies were low, and had no high association with neurologic symptoms and MRI changes. Intavenous methylprednisone and further cyclophosphamide therapy was ineffective and Rituximab – a chimeric anti-CD20 monoclonal antibody, as anti-viral tratment - has been used effectivelly. Her lupus-nefritis disappeared.

## Conclusions

Neuropsychiatric disturbance due to CNS lupus must be a diagnosis of exclusion, especially in immunosuprressed patients. All other possible causes of neurologic and psychiatryic symptoms. particularly viral infection must be considered. Rituximab was effective in treatment of Epstein-Bar encephalitis and improved symptoms of severe SLE.

